# Global proteomic analysis deciphers the mechanism of action of plant derived oleic acid against *Candida albicans* virulence and biofilm formation

**DOI:** 10.1038/s41598-020-61918-y

**Published:** 2020-03-20

**Authors:** Subramanian Muthamil, Krishnan Ganesh Prasath, Arumugam Priya, Pitchai Precilla, Shunmugiah Karutha Pandian

**Affiliations:** Department of Biotechnology Science Campus Alagappa University Karaikudi, 630 003 Tamil Nadu, India

**Keywords:** Mass spectrometry, Biofilms

## Abstract

*Candida albicans* is a commensal fungus in humans, mostly found on the mucosal surfaces of the mouth, gut, vagina and skin. Incidence of ever increasing invasive candidiasis in immunocompromised patients, alarming occurrence of antifungal resistance and insufficient diagnostic methods demand more focused research into *C. albicans* pathogenicity. Consequently, in the present study, oleic acid from *Murraya koenigii* was shown to have the efficacy to inhibit biofilm formation and virulence of *Candida* spp. Results of *in vitro* virulence assays and gene expression analysis, impelled to study the protein targets which are involved in the molecular pathways of *C. albicans* pathogenicity. Proteomic studies of differentially expressed proteins reveals that oleic acid induces oxidative stress responses and mainly targets the proteins involved in glucose metabolism, ergosterol biosynthesis, lipase production, iron homeostasis and amino acid biosynthesis. The current study emphasizes anti-virulent potential of oleic acid which can be used as a therapeutic agent to treat *Candida* infections.

## Introduction

*Candida* is a genus of yeast with remarkable phenotypic characteristics and found as a commensal fungus in humans. *Candida* species are most commonly present in the genital tracts and other membrane tracts such as mucosal oral cavity, respiratory tract, gastrointestinal tract etc^1^. Although over hundred *Candida* species belong to this genus*, C. albicans* is responsible for the majority of *Candida* infection. Other medically important *Candida* species are *Candida glabrata, Candida tropicalis, Candida dubliniensis*, and *Candida parapsilosis*^2^. Also, *C. albicans* is able to form well structured, three dimensional biofilms comprising of round yeast cells, filamentous hyphae, pseudohyphae and exopolysaccharides which prevent the action of antifungal agents and safeguard the pathogen from host defense mechanism^3^. While most of the implant associated infections are caused by *C. albicans* biofilm, few non *C. albicans Candida* species (NCAC) including *C. glabrata* and *C. tropicalis* have also been reported for their participation in urinary tract and blood stream infections^4^. Worldwide, candidiasis is the fourth most healthcare associated infection in hospitalized patients and the pathogen *Candida* is well known for its device associated infection. In general, gold standard antifungal agents such as azoles, amphotericin B, polyenes and flucytosine are most frequently used for the treatment of *Candida* antifungal therapy. However, extensive usage of these antifungal agents makes the pathogen develop resistance against these drugs. In recent years, increased usage of these antimycotics in antifungal therapies, transplantation, AIDS and diabetes are the major factors of *C. albicans* infections in hospitalized patients. The pathogenic nature of *Candida* species is regulated by virulence traits such as morphological transition, contact sensing, biofilm development, invasion, adhesion on the cell surface and hydrolytic enzyme secretion^5^. To overcome these drug resistant and biofilm mediated *Candida* infections, there is an immediate requirement of alternative anti-pathogenic agents.

Though traditionally medicinal plants are extensively used for the treatment of many diseases, it is estimated that only 1–10% of ~250,000–500,000 plants on Earth are being used by humans^6^. In recent decades, medicinal plants have been widely reported for their antimicrobial effect against various bacterial and fungal pathogens. In addition, plants are also used as food preservatives, dietary supplements, food spoilage, flavor enhancers, etc. The major advantages of using plant-derived compounds as therapeutic agents are less adverse effects, multiple mode of action and low chances of antimicrobial resistance^7^. Our research group has recently reported the anti-infective potential of several phytocompounds against bacterial and fungal pathogens. For instance, 3-Furancarboxaldehyde and limonene against Group A Streptococcus^8,9^, 2-Furaldehyde diethyl acetal (*Cocos nucifera*), curcumin and other phytocompounds against *Pseudomonas aeuginosa*^10,11^, 3-*O*-methyl ellagic acid (*Anethum graveolens*) and vanillic acid (*Actinidia deliciosa*) against *Serratia marcescens*^12,13^, Embelin (*Emblica ribes*) in combination with ketoconazole against *Malassezia* spp.^14^, and synergistic combination of quinic acid and undecanoic acid against *Candida* spp^15^. In addition, oleic acid has been reported for its antibacterial and antifungal activity against various Gram positive and Gram negative bacterial pathogens and fungal pathogens^16,17^. However, reports are scanty on the mechanism of action of oleic acid. In this backdrop, the present study aimed to explore the anti-virulence efficacy of oleic acid derived from *Murraya koenigii* against *Candida* spp. through transcriptomic and proteomic approaches.

## Results

### Oleic acid disassembles *Candida* spp. biofilm

**Figure 1:**
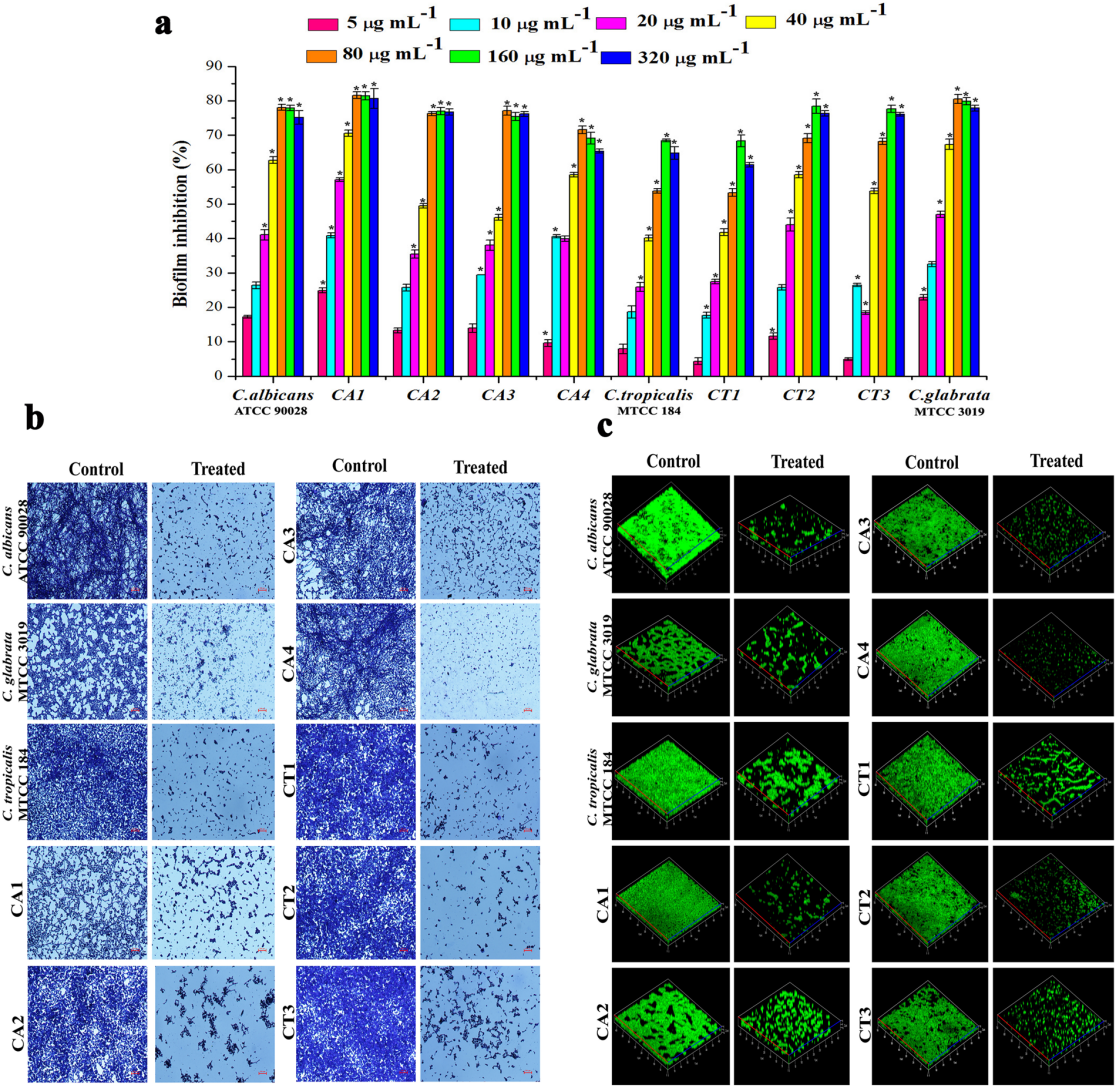
Antibiofilm activity of oleic acid against *Candida* spp. without affecting fungal growth and viability. **(a)** Biofilm inhibitory potential of oleic acid against *Candida* spp. in a dose dependent manner in spider broth at 37 °C for 24 h. **(b)** Light microscopic images depicting *Candida* spp. biofilm formed on glass surfaces in the absence and presence of oleic acid in spider broth at 37 °C for 24 h. Magnification 400×, scale bar – 200 µm. **(c)** 3D representation of biofilm dispersal induced by oleic acid in *Candida* spp. as visualized by confocal laser scanning microscopy.

To investigate the effect of oleic acid on *Candida* spp. and to determine the biofilm inhibitory concentration (BIC), standard crystal violet quantification method was used. The results of antibiofilm assay showed a concentration dependent increase in biofilm inhibition. BIC of oleic acid was varying between *Candida* species. For the wild type (ATCC 90028) and clinical isolates of *C. albicans* (CA1, CA2, CA3 and CA4) and *C. glabrata* (MTCC 3019), BIC was found to be 80 µg mL^−1^ (Fig. 1a). Whereas, BIC of *C. tropicalis* (MTCC184), and the clinical isolates (CT1, CT2 and CT3) was found to be 160 µg mL^−1^.

### Oleic acid disassembles *Candida* spp. biofilm without affecting fungal growth and viability

Oleic acid did not inhibit the growth of all the tested *Candida* strains at varying concentrations (10 µg mL^−1^ to 640 µg mL^−1^) confirming the non-fungicidal antibiofilm effect of oleic acid (Supplementary Fig. S1a). Also, this result was further authenticated by XTT assay wherein the viability of oleic acid treated cells was similar to that of control samples in all the tested strains (Supplementary Fig. S1b).

### Microscopic visualization confirms biofilm disassembly of oleic acid

To evaluate the antibiofilm efficacy of oleic acid on glass surface, light microscopic analysis was performed. In the light micrographs, control samples were seen with thick layer of biofilm matrix consisting of yeast and hyphae cells. Whereas microcolony formation and hyphal elongation was completely inhibited by oleic acid in treated samples (Fig. 1b). To further corroborate the antibiofilm activity of oleic acid, CLSM analysis was performed. Three-dimensional view of *Candida* spp. biofilm clearly revealed reduction in the biofilm thickness and biomass and mature biofilm architecture in all the tested slides when compared to their respective controls (Fig. 1c).

### Oleic acid controls filamentous growth of *Candida* spp

**Figure 2:**
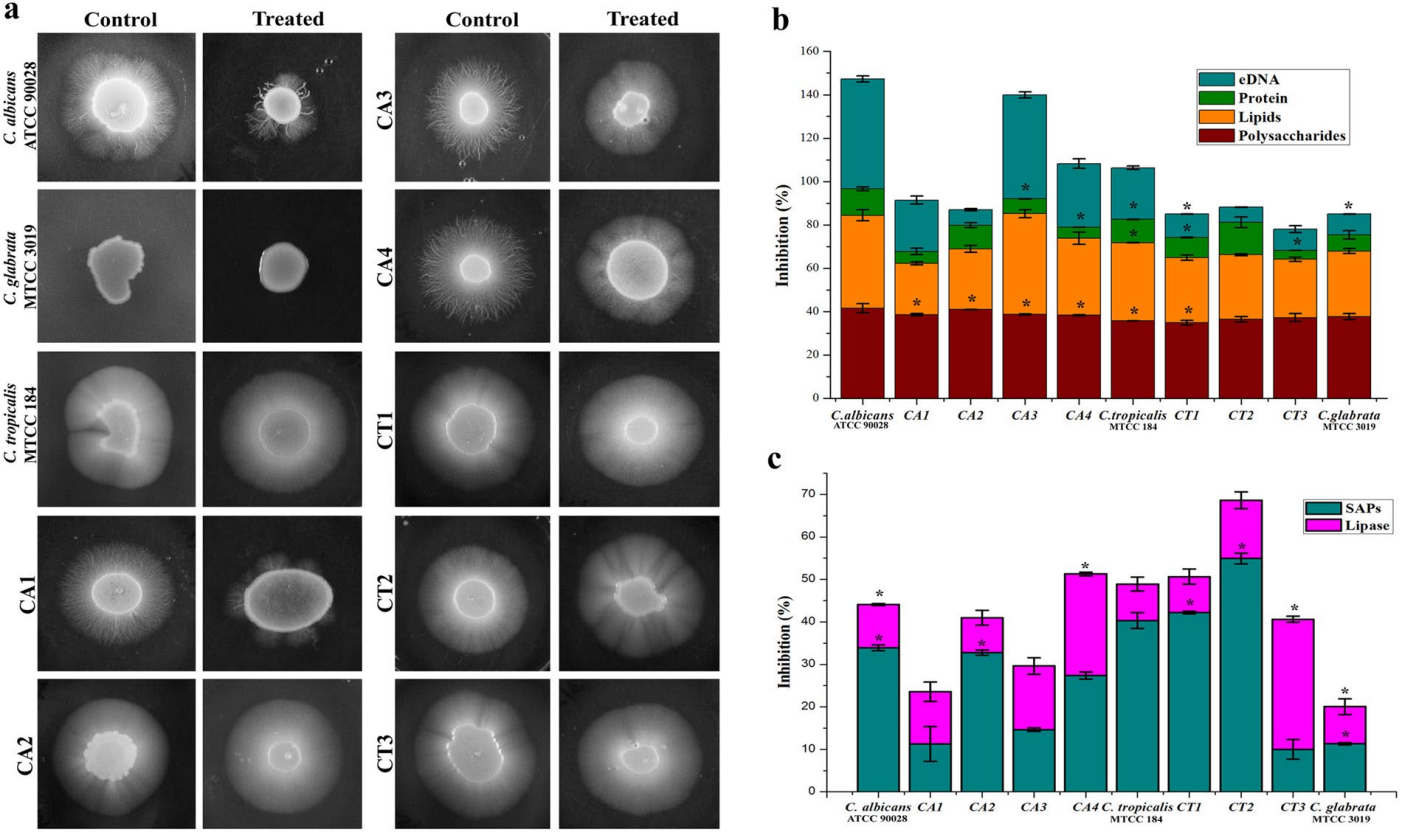
(**a**) Oleic acid inhibiting filamentous growth of *Candida* spp. in spider agar supplemented with 10% FBS. Oleic acid strongly reduced the filamentation ability in *C. albicans* (ATCC 90028) and clinical isolates (CA1, CA2, CA3 and CA4). Moderate reduction in the filamentous growth was observed in *C. tropicalis* (MTCC 184) and its clinical isolates (CT1, CT2, CT3). No filamentous growth was observed in *C. glabrata* (MTCC 3019). **(b)** Quantification of EPS components such as polysaccharides, lipids, proteins and eDNA extracted from *Candida* spp. biofilm matrix by phenol sulfuric acid method (absorbance at 490 nm), phospho-vanillin method (absorbance at 545 nm), Bradford method (absorbance at 595 nm) and nano spectrophotometer (absorbance at 260/280 ratio), respectively. Oleic acid mainly inhibits the polysaccharides and lipids present in the biofilm matrix. **(c)** Effect of oleic acid on secreted aspartyl proteinases (SAPs) and lipases produced by *Candida* spp. using bovine serum albumin agar and lipase agar, respectively. Error bars represent standard deviations from the mean (n = 3). Statistical significance was analyzed using one way ANOVA-Duncan’s post-hoc test and single asterisk represent p < 0.05.

Effect of oleic acid on *Candida* spp. filamentation was assessed using spider agar supplemented with 10% FBS. In the case of *C. albicans* wild type strain (ATCC 90028) and the clinical isolates (CA1, CA2, CA3 and CA4), filamentous growth was significantly inhibited upon oleic acid treatment (Fig. 2a). On the other hand, in *C. tropicalis* (MTCC) and its isolates (CT1, CT2 and CT3) only slight inhibition in the hyphal growth was noticed. *C. glabrata* is hyphal negative, thus no filamentous growth was observed even after 72 h of incubation.

### Oleic acid disperses biofilm matrix enclosed by extra polymeric substances (EPS)

The EPS of *Candida* spp. encompasses three-dimensional structure of yeast, hyphae, pseudohyphae and biomolecules such as carbohydrates, proteins, lipids and nucleic acids^15^. In all the tested *Candida* strains, polysaccharides and lipids present in the EPS were decreased up to 35–41% and 26–47%, respectively (Fig. 2b). Moreover, slight inhibition (4–14%) was observed in the protein level of EPS upon oleic acid treatment. Intriguingly, oleic acid was found to inhibit eDNA level of EPS in an uneven manner, wherein a maximum of 47% inhibition was observed in CA3 and only 6% inhibition was noticed in CT3 strain.

### Oleic acid reduces secreted hydrolases production in *Candida* spp

Effect of oleic acid on *Candida* spp. secreted aspartyl proteinases (SAPs) and lipase production was qualitatively measured using BSA and tributyrin agar, respectively^15^. White precipitation zone around the colonies indicates the SAPs production. Zone diameter was measured for both control and treated plates and the percentage of inhibition was calculated. Likewise, lipase production was also quantitatively measured by zone of clearance and the percentage of inhibition was measured. In all the tested strains, production of SAPs and lipases was inhibited up to 10–54% and 8–30%, respectively (Fig. 2c).

### Oleic acid modifies ergosterol constituent of fungal cell membrane

**Figure 3:**
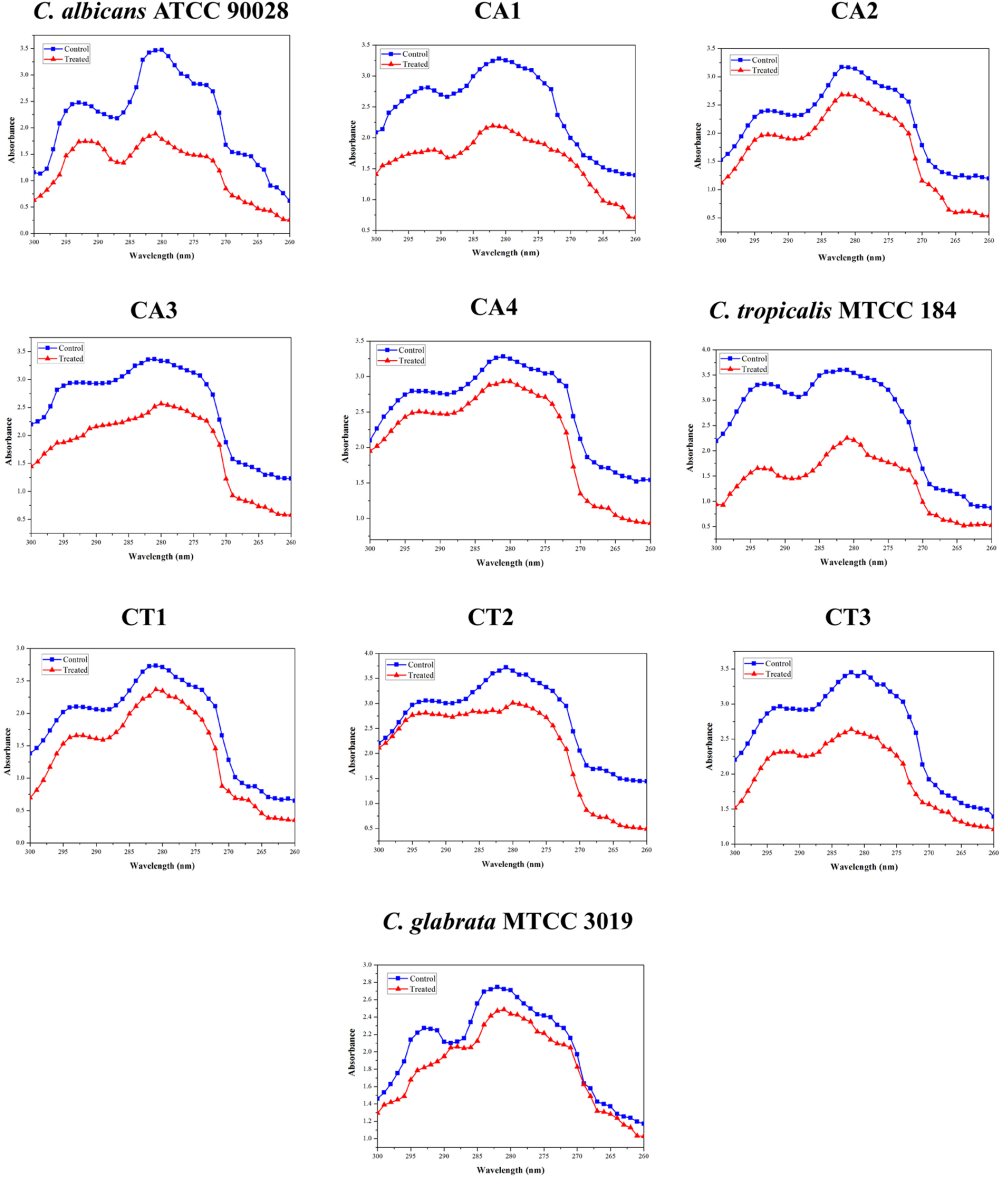
UV spectra revealing the effect of oleic acid on ergosterol profile of *Candida* spp. Ergosterol present in *Candida* spp. with and without oleic acid scanned from 260 to 300 nm. Reduction in the peak height represents changes in ergosterol content. UV scanning spectra represented four peaks between 260–300 nm corresponding to ergosterol and sterol intermediates.

Ergosterol is an important component of fungal cell membrane maintaining the integrity, permeability and structure^3^. Hence, the change in the ergosterol production upon treatment with oleic acid was evaluated using UV spectrophotometer. UV scanning spectra represented four peaks between 260–300 nm corresponding to ergosterol and sterol intermediates and the flat line without peaks indicated the absence of sterol. As can be seen from Fig. 3, Oleic acid drastically reduced the ergosterol content of *C. albicans, C. tropicalis*, CA1, CA3 and CT3 strains and in the remaining *Candida* strains moderate ergosterol inhibition was noticed.

### Effect of oleic acid on *C. albicans* adhesion ability and H_2_O_2_ sensitivity

**Figure 4:**
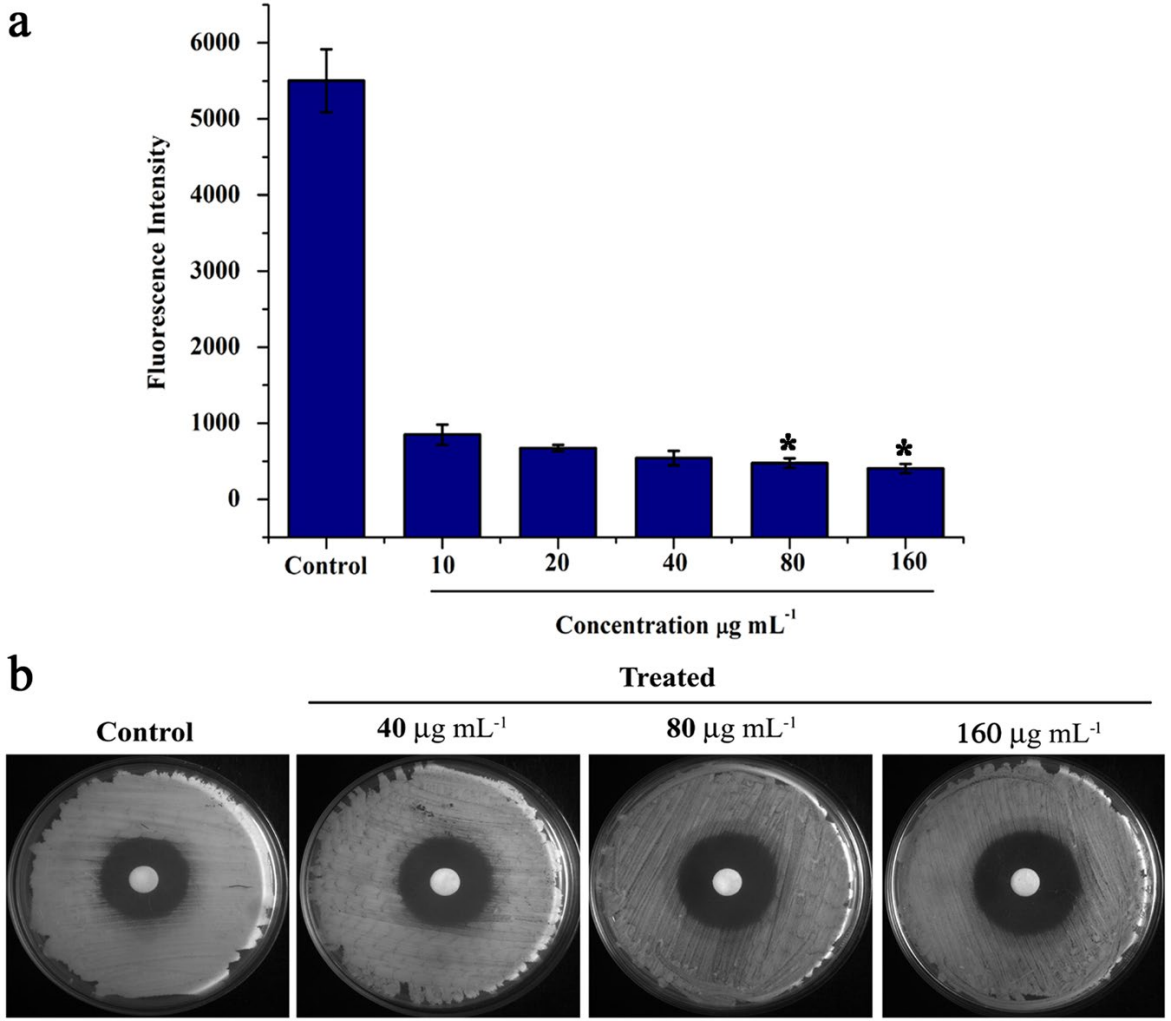
Effect of oleic acid *C. albicans* adhesion ability and H_2_O_2_ sensitivity. **(a)** Alamar blue reduction assay was performed to check the effect of oleic acid on *C. albicans* adhesion ability to the polystyrene surfaces. Fluorescence intensity dose dependently decreased in oleic acid treated samples compared to control. **(b)** H_2_O_2_ sensitivity assay - Representative YEPD agar plates depicting the effect of oleic acid treatment on the sensitivity of *C. albicans* to H_2_O_2_. Error bars represent standard deviations from the mean (n = 3). Statistical significance was analyzed using one way ANOVA-Duncan’s post-hoc test and single asterisk represent p < 0.05.

The very first event of *Candida* biofilm formation is the adhesion to host surfaces for both colonization and establishment of infections. Thus, effect of oleic acid on *C. albicans* adherence to the polystyrene surfaces was assessed using alamar blue assay. The fluorescent intensity of alamar blue or resazurin dye was found to be decreased in a dose dependent manner in oleic acid treated samples when compared to control. This result clearly depicts the anti-adhesion ability of oleic acid on *C. albicans* biofilm attached to the polystyrene surfaces (Fig. 4a).

Results of H_2_O_2_ sensitivity assay showed that oleic acid treated *C. albicans* cells were more sensitive to H_2_O_2_ than control. Briefly, in control plates, zone of clearance around H_2_O_2_ was measured as 32 mm (Fig. 4b), whereas in the case of 40, 80 and 160 µg mL^−1^ of oleic acid treated plates, sensitivity to H_2_O_2_ were increased with the diameter of 34, 36 and 38 mm respectively (Fig. 4b).

### Impact of oleic acid on *C. albicans* virulence gene expression

**Figure 5:**
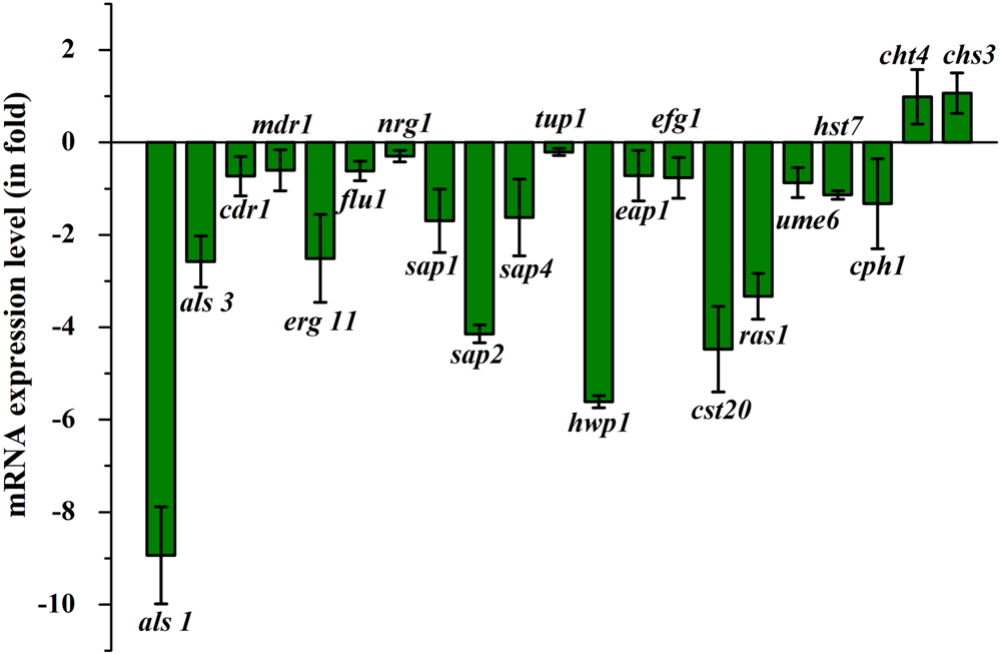
Gene expression profile of *C. albicans*. Impact of oleic acid on the expression of candidate genes involved in virulence factors (*als1, als3* - adhesion, *cdr1, mdr1* – efflux pump mechanism, *erg11* - ergosterol production, *flu1* – fluconazole resistance gene, *sap1, sap2, sap4* - SAPs production, *hwp1* - hyphal elongation, *nrg1, tup1* – negative regulators of transcription, *cst20, hst7* – filamentation, *efg1, cph1* – transcription factor, *ume6* - Transcriptional regulator of filamentous growth, *ras1, eap1* – cell adhesion, *chs3* – synthesis of chitin, *cht4* – degradation of chitin). Relative gene expression level was determined using the ΔΔCT method. Error bars represent standard deviations from the mean (n = 3).

Quantitative PCR was used to assess the inhibitory effect of oleic acid on the transcriptional regulatory network of biofilm formation and virulence of *C. albicans*. Among the tested genes, candidate genes involved in adhesion (*als1*), SAPs production (*sap2*), hyphal elongation (*hwp1*) and filamentation (*cst20*) were significantly down regulated up to 8.9, 4.1, 5.6 and 4.4 fold respectively. In addition, genes involved in ergosterol production (*erg11* – 2.5 fold), cell adhesion (*ras1* – 3.3 fold) and hyphal transcription factor (*cph1-* 1.3 fold) were moderately down regulated by oleic acid. Besides, oleic acid slightly down regulated (<1.0 fold) the expression of other genes. But, the regulation of cell wall related genes chitin synthase-3 (*chs3* – 1.0 fold) and chitinase (*cht4* – 0.9 fold) were found to be slightly upregulated (Fig. 5).

### Effect of oleic acid treatment on the cellular proteome of *C. albicans*

**Figure 6:**
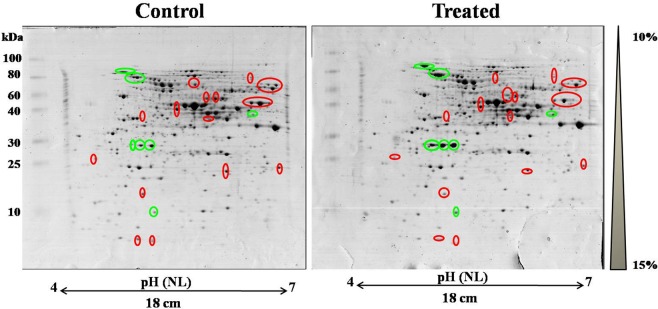
Effect of oleic acid on intracellular proteome of *C. albicans*. Representative gel images depicting the comparison between intracellular protein extract of *C. albicans* grown in the absence and presence of 80 µg mL^−1^ of oleic acid by 2- D gel electrophoresis. One thousand microgram of protein was subjected to isoelectric focusing and resolved in 10–15% gradient SDS-PAGE and the protein spots were stained with MS compatible CBB G-250. Up regulated and down regulated protein spots are highlighted in green and red circles, respectively.

**Figure 7:**
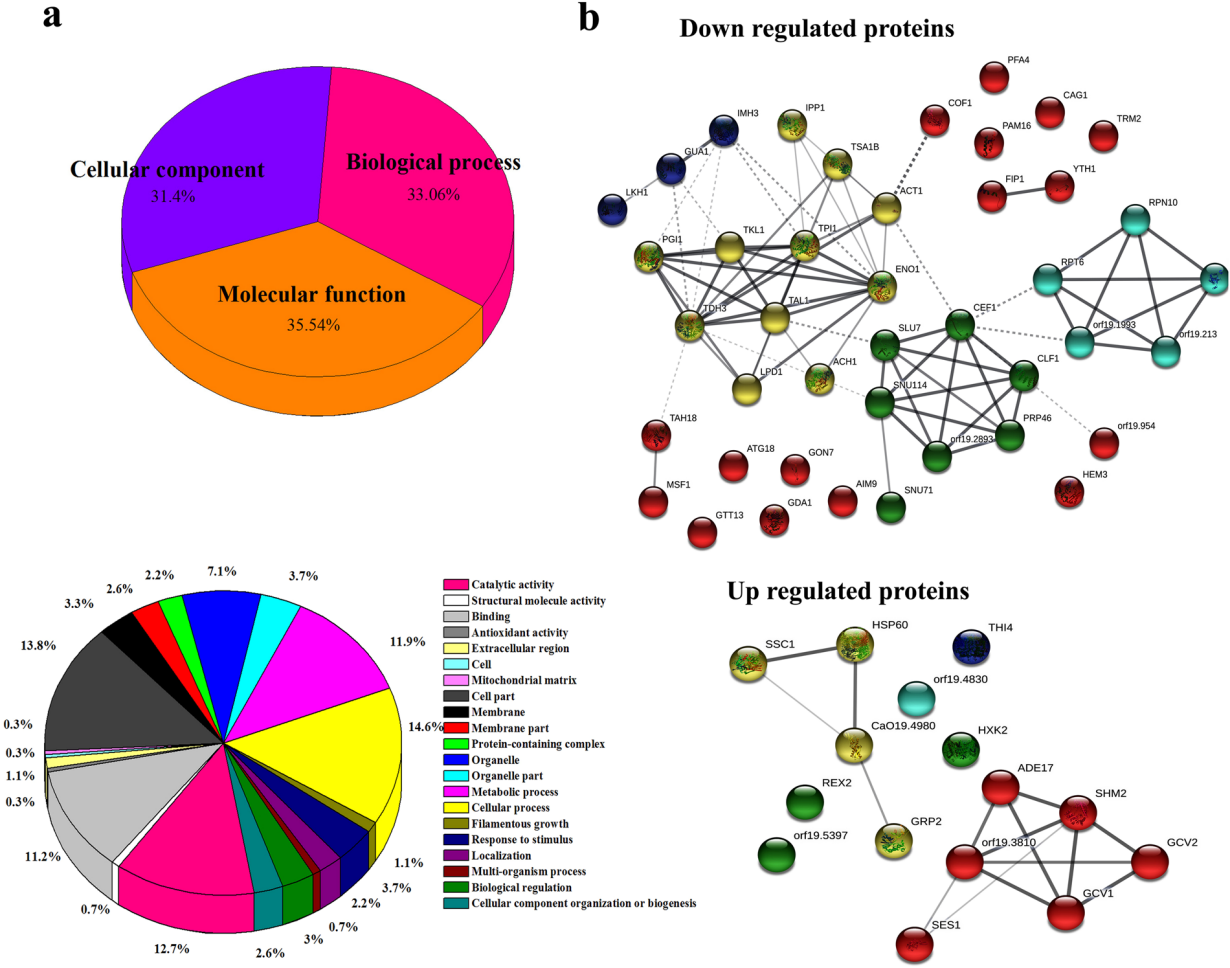
Gene ontology and STRING analysis of differentially regulated proteins of *C. albicans* upon oleic acid treatment. **(a)** Gene ontology analysis of differentially regulated (both up regulated and down regulated) proteins using UniProt database. The differentially expressed proteins are mainly involved in cellular process (37%), catalytic activity (33%), metabolic process (30%), binding (29%), response to stimulus (9%), biological regulation (8%), and cellular component organization (7%) **(b)** Protein-protein interaction prediction map of (i) down regulated and (ii) up regulated proteins of *C. albicans* obtained using STRING v.11 database (confidence score 0.400). The interaction map shows the proteins involved in interrelated pathways such as proteasome, spliceosome, carbon metabolism, biosynthesis of antibiotics, biosynthesis of secondary metabolites and metabolic pathways. Filled protein nodes that signify the availability of protein 3D structural information is known or predicted.

To find out the mechanism of action of oleic acid on *C. albicans* virulence, two dimensional gel electrophoresis (2D-PAGE) coupled with mass spectrometry (MALDI-TOF/TOF) technique was used. Intracellular proteins from *C. albicans* grown (16 h) in the absence and presence of oleic acid were extracted by sonication. The extracted proteins were quantified using standard Bradford method and SDS-PAGE was performed in order to check the quality of protein and identify the differential expression between control and treated samples. Afterwards, cellular proteome of control and oleic acid treated *C. albicans* was assessed using 2D-PAGE (Fig. 6). Image Master Platinum software (Version- 7.0, GE Healthcare, USA) was used to detect and match the protein spots present in the control and treated gels. Based on the results obtained from densitometric analysis, among the 300 detected spots, eleven and forty spots were found to be up regulated and down regulated (>1.5 fold), respectively. Based on statistical significance (ANOVA - P < 0.05) and differential expression (≥1.5 fold), 51 spots were selected for protein identification and subjected to MALDI-TOF/TOF analysis. Then, the obtained MALDI spectra were matched with Swissprot.2017.11.01 database using MS-Fit online software tool. The details of differentially regulated proteins and their function, number of peptides matched, sequence coverage and MOWSE score are listed in Table 1. In addition, gene ontology analysis in UniProt was used to identify the functions of differentially regulated proteins. From this analysis it became evident that the differentially expressed proteins are mainly involved in cellular process (37%), catalytic activity (33%), metabolic process (30%), binding (29%), response to stimulus (9%), biological regulation (8%), and cellular component organization (7%) (Fig. 7a). Furthermore, protein-protein interaction among differentially regulated proteins (both up regulated and down regulated proteins) was done using STRING v.11 database with confidence score (0.4) and the resultant protein-protein interaction map is shown in Fig. 7b. These results suggest that the differentially regulated proteins are predominantly involved in major pathways including proteasome, spliceosome, carbon metabolism, biosynthesis of antibiotics, biosynthesis of secondary metabolites and metabolic pathways.

**Table 1:** List of differentially expressed proteins of *C. albicans* treated with oleic acid identified using MALDI-TOF/TOF.

Spot ID	Fold	P-value	Accession No.	Description	MOWSE score	Coverage (%)	No. of Peptides matched	Gene Name
**Down regulated proteins**								
CAP1	4.2	0.002	P83773	Acetyl-CoA hydrolase	2.86E + 11	19.7	20	*ACH1*
CAP3	3.2	0.006	P43067	Alcohol dehydrogenase	184	6.3	7	*ADH1*
CAP5	2.8	0.003	O94048	Porphobilinogen deaminase	1195	16.8	12	*HEM3*
CAP6	2.8	0.152	Q59T36	mRNA 3′-end-processing protein	6.41E + 06	30	15	*YTH1*
CAP8	2.6	0.003	O74713	High-affinity glucose transporter	4413	8.6	10	*HGT1*
CAP10	2.4	0.036	Q59W04	EKC/KEOPS complex subunit	10310	33.1	7	*GON7*
CAP11	2.4	0.001	Q5APF2	GMP synthase [glutamine-hydrolyzing]	32644	20.6	9	*GUA1*
CAP12	2.3	0.004	P34725	Phospho-2-dehydro-3-deoxyheptonate aldolase, phenylalanine-inhibited	1382	8.2	6	*ARO3*
CAP16	2.0	0.006	Q5A4Z2	Uncharacterized protein	1025	21	13	*orf19.954*
CAP17	2.0	0.238	Q92211	Glyceraldehyde-3-phosphate dehydrogenase	39759	23	12	*TDH1*
CAP19	2.0	0.002	Q0ZIB4	Mitochondrial GTPase elongation factor Tu (Fragment)	902	32.6	4	*TUF1*
CAP20	2.0	0.000	Q5AM84	U1 small nuclear ribonucleoprotein component	952	8.1	12	*SNU71*
CAP21	2.0	0.044	Q8TGH6	Guanosine-diphosphatase	489	13.4	8	*GDA1*
CAP24	1.9	0.000	A0A1D8PMW6	Cofilin	3093	59.6	8	*COF1*
CAP26	1.9	0.000	P28868	Guanine nucleotide-binding protein	1943	9.3	7	*CAG1*
CAP29	1.8	0.043	Q5AD28	Phenylalanine–tRNA ligase	94591	17.7	9	*MSF1*
CAP30	1.8	0.000	Q59X29	Proteasome regulatory particle base subunit	1204	11.1	4	*RPN10*
CAP31	1.8	0.130	Q02751	Alpha-glucosidase	4802	19.8	7	*MAL2*
CAP32	1.8	0.000	A0A1D8PCG3	Trm2p	46991	16	10	*TRM2*
CAP34	1.7	0.004	Q0P7I3	Dit1 protein	661	11.2	7	*DIT1*
CAP36	1.7	0.052	Q5ADL4	Pre-mRNA-splicing factor	1.92E + 06	15.7	12	*SLU7*
CAP37	1.7	0.002	Q5A922	Altered inheritance of mitochondria protein 9	2030	14.9	10	*AIM9*
CAP38	1.7	0.000	Q8J224	Alpha subunit of farnesyl transferase	210	14.7	4	*RAM2*
CAP40	1.7	0.001	P83777	Inorganic pyrophosphatase	984	14.6	6	*IPP1*
CAP44	1.6	0.002	Q5A750	Transketolase	836518	15.7	10	*TKL1*
CAP46	1.6	0.007	Q5AD27	NADPH-dependent diflavin oxidoreductase 1	14468	13.1	8	*TAH18*
CAP47	1.6	0.001	G1UA34	Putative uracil phosphoribosyltransferase	5054	33.5	10	*FUR1*
CAP48	1.6	0.002	P78594	Cytosine deaminase	90713	11.3	6	*FCA1*
CAP50	1.6	0.015	A0A1D8PJQ3	Gtt13p	10203	12.2	11	*GTT13*
CAP51	1.6	0.018	Q5ABA6	Autophagy-related protein 18	12699	17.2	10	*ATG18*
CAP53	1.6	0.006	Q59ZW9	Mitochondrial import inner membrane translocase subunit	2059	22.3	6	*PAM16*
CAP54	1.6	0.004	A0A1D8PFR4	Actin	91474	26.1	12	*ACT1*
CAP55	1.6	0.048	G1UAT0	Uncharacterized protein	1676	9.1	12	*CaJ7.0419*
CAP58	1.5	0.001	P78589	Squalene synthase	502	12.3	10	*ERG9*
CAP59	1.5	0.002	Q59RQ6	Dihydrolipoyl dehydrogenase	203000000	21.2	16	*LPD1*
CAP60	1.5	0.000	Q59NB8	Leukotriene A-4 hydrolase homolog	576307	15.4	13	*LKH1*
CAP62	1.5	0.002	Q5AGV7	Palmitoyltransferase	56899	11.9	11	*PFA4*
CAP63	1.5	0.046	O94091	Lipase 1	1808	10.9	5	*LIP1*
CAP64	1.5	0.067	Q9HFV5	1-(5-phosphoribosyl)-5-[(5-phosphoribosylamino)methylideneamino] imidazole-4-carboxamide isomerase	5199	15.4	6	*HIS6*
CAP65	1.5	0.003	P0CU34	Peroxiredoxin	36223	25.5	7	*TSA1B*
**Up regulated proteins**								
CAP2	3.2	0.001	Q5A313	Uncharacterized protein	580	9.7	7	*CAALFM_CR08740WA*
CAP4	3.0	0.000	A0A1D8PGG3	Rex2p	23793	27.8	12	*REX2*
CAP7	1.9	0.410	O13426	Serine hydroxymethyltransferase	7.85E + 03	18.7	8	*SHM2*
CAP13	2.3	0.002	C4YNP4	Thiamine thiazole synthase	2.50E + 10	37	22	*THI4*
CAP15	2.1	0.002	Q5A7S0	Golgi transport complex subunit	4491	16.9	9	*orf19.5397*
CAP25	1.9	0.017	P83776	Hexokinase-2	732657	27.7	13	*HXK2*
CAP28	1.8	0.002	Q9HGT6	Serine–tRNA ligase, cytoplasmic	375	13.2	11	*SES1*
CAP43	1.6	0.052	Q5APD5	Uncharacterized protein	28945	18.2	5	*orf19.4830*
CAP45	1.6	0.000	G1UAH4	Uncharacterized protein	1405	20.8	14	*CaJ7.0423*
CAP52	1.6	0.037	P41797	Heat shock protein SSA1	5056	26.5	13	*SSA1*
CAP67	1.5	0.022	P83784	Heat shock protein SSC1	390937	17.3	11	*SSC1*

## Discussion

Historically, *Candida* spp., *Cryptococcus* spp., *Aspergillus* spp. and *Fusarium* spp. have been reported as opportunistic fungal pathogens in hospitalized patients^18^. Candidiasis is the recurrent fungal infection caused by *Candida* genus, primarily *C. albicans* followed by *C. parapsilosis*, *C. glabrata*, *C. tropicalis* and *C. krusei*^19^. Although, emergence of antifungal resistance to conventional antifungal drugs is the major challenge to research scientists and pharmaceutical companies^20^. Recently published reports have suggested that plant derived compounds or phytocompounds can certainly be more efficient in controlling biofilm related infections^21–23^. Furthermore, the chances of drug resistance and adverse effects of these plant derived compounds are very low than conventional antibiotics or antifungal agents^12^. In this context, the present study established the inhibitory potential of oleic acid derived from *M. koenigii* against *Candida* spp. biofilm and virulence through *in vitro* assays, gene expression studies and proteomic approaches.

Primarily, Biofilm inhibitory concentration (BIC) of oleic acid was determined for all the tested *Candida* strains and exhibited maximum of 80% biofilm inhibition. Likewise, oleic acid derived from *Withania somnifera* and oleic acid coated magnetic nanoparticles exhibited substantial antibiofilm activity against various bacterial pathogens^24,25^. Previous studies revealed the antimicrobial activity of oleic acid against certain bacterial and fungal pathogens^17,26^. However, according to Rasmussen and Givskov (2006)^27–29^, compounds or antibiotics that kill or inhibit the growth of microorganism situates an organism under selection pressure to develop drug resistance. Hence, antifungal effect of oleic acid was assessed using broth dilution assay and the results clearly suggested that oleic acid did not inhibit the growth of *Candida* spp. XTT assay was performed to corroborate the non-fungicidal effect of oleic acid against *Candida* spp. The obtained results manifestly proved that the oleic acid does not inhibit the metabolic viability of *Candida* strains. Besides, microscopic techniques such as light microscopy and CLSM further demonstrated the inhibitory effect of oleic acid on *Candida* spp. biofilm formation and yeast to hyphal transition. It is akin to mammalian apoptosis inducer BH3l-1 and its derivatives effectively blocking the yeast-to-hyphal and yeast-to-pseudohyphal transitions in *C. albicans*^30^.

In *Candida* spp., filamentous growth is influenced by several factors including glucose and nitrogen starvation, oxygen deficiency, presence of N-acetylglucosamine and serum^31^. The filamentation assay results evidently showed that oleic acid significantly inhibits the filamentous growth of wild type *C. albicans* and *C. tropicalis*. However, trivial inhibition was observed in the filamentous growth of clinical isolates of *Candida* spp. This result is in agreement with an earlier study made by Manoharan *et al*.^32^, wherein, 7-benzyloxyindole (indole derivative) considerably inhibited the filamentous growth of *C. albicans* even after 10 days of incubation. In the case of *Candida* biofilm, EPS matrix furnishes protective environment to the biofilm cells from host immune system and from the antifungal agents^3,15^. Hence, quantification of EPS components was carried out which revealed that oleic acid treatment could significantly reduce the carbohydrates, lipids and eDNA content of EPS. However, no considerable change was detected in the protein level of EPS. In the same way, compounds from natural resources such as 2,4-di-tert-butylphenol from *Vibrio alginolyticus* G16, usnic acid from lichen and 5-hydroxymethyl-2-furaldehyde from *Bacillus subtilis* efficiently disrupted the EPS of *C. albicans*^33–35^. Secreted hydrolases such as SAPs, phospholipases and lipases play a significant role in *Candida* spp. pathogenicity and these enzymes facilitate the invasion of hyphal cells into host tissues^36,37^. SAPs and lipase production of *Candida* spp. were qualitatively measured, while oleic acid moderately inhibited SAPs production. Previously, mycogenic AgNPs have been shown to exhibit significant inhibition against SAPs production and biofilm growth in *C. albicans* and NCAC species^38^. On the other hand, only slight inhibition was noticed in lipase production. Enzymes involved in ergosterol pathway are the main target of most of the antifungal agents including azoles and polyenes. Besides ergosterol, membrane lipid modulates cell membrane fluidity, permeability, and integrity^15,39,40^. In the tested *Candida* strains, oleic acid produced substantial changes in the ergosterol content of each *Candida* spp. which undoubtedly suggest that oleic acid could be a potent drug than conventional antifungal agents. Similarly, Masood *et al*.^41^ have reported that a novel series of 1,2,3-triazole–quinazolinone conjugates interferes with sterol biosynthetic pathway and decreases the ergosterol content in *C. albicans*. Adhesion to biotic or abiotic surfaces is the first step of *C. albicans* biofilm formation^15^. Results of alamar blue assay clearly indicate that oleic acid significantly inhibited the adhesion ability of *C. albicans* cells to the polystyrene surfaces in a concentration dependent manner. *In vitro* assay results further substantiated at the transcription level using real time PCR. Oleic acid treatment considerably down regulated the genes involved in virulence of *Candida* spp., such as adhesion (*als1, als3, eap1*), ergosterol biosynthesis (*erg11*), SAPs production (*sap1, sap2, sap4*), filamentation (*hwp1, efg1, cst20*, *ras1, ume6, hst7*) and efflux pump mechanism (*cdr1, mdr1*). In a similar way, synergistic combination of quinic acid and undecanoic acid significantly down regulated the major virulence genes of *C. albicans*^15^. Yet another study reported that linalool, terpene alcohol, could inhibit yeast-hyphal transition through cAMP-PKA pathway (via *efg1* gene), MAPK pathway (via *cph1* gene), control adhesins (*als3* gene) and hyphal maintanence (*eed1, ume6, hgc1*) thereby persuading the *C. albicans* biofilm formation^42^. Conversely, oleic acid did not show any negative impact on the genes chitin synthase-3 (*chs3*) and chitinase-4 (*cht4*) which are essential for cell wall strength and flexibility, respectively^43^.

In the current study, 51 proteins were identified by MALDI-TOF/TOF followed by MS-Fit analysis. Based on proteins’ function and their importance in *C. albicans* virulence, 20 differentially regulated proteins were discussed in this study. Proteomic analysis revealed that oleic acid significantly down regulated the expression of acetyl-coA hydrolase (Ach1p) by 4.2 fold. pH homeostasis plays an important role in virulence mechanism of *C. albicans* in various host environments and the pathogen is capable of changing acidic/alkaline environment to neutral pH. Neutral pH promotes the morphological transition of yeast to hyphae which enhance the virulence of pathogen. This sort of pH adaptation is related to aminoacids catabolism by the enzyme acetyl-coA hydrolase and urea amidolyase (Dur1)^44,45^. The down regulation of Ach1p by oleic acid is comparable to physiological assays, wherein, microscopic visualization and filamentation assay results clearly proved that oleic acid considerably inhibited the hyphal growth of *C. albicans* in spider medium (at pH 7.2).

Alcohol dehydrogenase enzyme (Adh1) catalyzes both ethanol fermentation and metabolism. Adh1 is one of the factors which controls planktonic growth and biofilm formation of *C. albicans* through an ethanol dependent mechanism. Further, Adh1 also regulates other virulence factors including yeast-to-hyphal transition, drug efflux mechanism^46–50^. In the present study, Adh1p expression was down regulated up to 3.2fold upon treatment with oleic acid. In *C. albicans*, there are three known iron uptake systems including iron utilization from hemoglobin^27,51,52^. Further, porpholinogen deaminase (Hem3) is an enzyme involved in heme biosynthetic pathway which gets induced in the presence of high iron and CO_2_ and the same was down regulated upto 2.8 fold by oleic acid treatment. Likewise, porpholinogen deaminase protects *Aspergillus nidulans* from reactive nitrogen species or nitrosative stress^53^.

In *C. albicans*, glucose majorly influences the yeast-to-hyphal transition and other virulence factors such as adhesion, biofilm formation, oxidative stress resistance, invasion, and antifungal drug tolerance^54,55^. Moreover, Glucose transporters are considered as major targets for antimicrobial drug development. In the present study, oleic acid treatment down regulated the expression of Hgt1 protein (2.6 fold) in YEPD + FBS medium. GMP synthase [glutamine-hydrolyzing] enzyme (GUA1) is involved in the subpathway of purine metabolism which synthesizes guanine 5’-monophosphate (GMP) from xanthine 5’-monophosphate (GMP). Earlier reports suggested that Gua1 could be a potent target for an antifungal compound which is essential for growth and virulence of *C. albicans*. An *in vivo* study results in murine model of systemic candidiasis clearly suggested that heterozygous mutant of Gua1 strain (*gua1/gua1*) was completely avirulent^56–58^. Down regulation of Gua1p in the current study clearly confirmed that oleic acid modulates the pathogenicity of *C. albicans*.

Phospho-2-dehydro-3-deoxyheptonate aldolase, phenylalanine-inhibited (Aro3), an enzyme involved in aromatic amino acid biosynthesis, is down regulated (2.3 fold) by oleic acid. Yin *et al*.^59^, reported the comparative response of general amino acid responses (GCN) of *S. cerevisiae* and *C. albicans* at the proteomic level. From the results, it is clear that Aro3p is increased in response to the histidine analogue, 3-aminotriazole, though oleic acid down regulated the expression of Aro3p. This report clearly suggests that *C. albicans* cells use amino acids as nitrogen source for their growth in host cells and amino acids utilization directly influences fungal pathogenesis by activating virulence factors such as biofilm growth and hyphal morphogenesis^60,61^. In the present study, oleic acid inhibited the hyphal morphology and biofilm formation, accordingly down regulated the expression of Aro3p.

In glycolysis pathway, glyceraldehyde-3-phosphate dehydrogenase or GAPDH (Tdh1) converts D-glyceraldehyde 3-phosphate into pyruvate. In previous report, Tdh1p expression was found to be up regulated in the presence of N-acetylglucosamine (GlcNAc) which induces morphological transition at amino acid depleted condition^62^. This enzyme Tdh1p expression was found to be 2.0 fold down regulated upon treatment with oleic acid. Guanosine diphosphatase or GDPase encoded by *GDA* is an enzyme involved in nucleotide sugar transport/antiport cycle from golgi apparatus to cytosol and it is present in yeast and hyphal forms of *C. albicans*. Null mutant strain *gda1/gda1* of *C. albicans* is severely defective in O-mannosylation and cell wall phosphate content which can slightly affect the hyphal growth^63–65^. Hyphal inhibitory effect of oleic acid could be the reason for the down regulation of Tdh1p and Gda1p.

In the present study, oleic acid down regulated the expression of alpha glucosidase (Mal2p) by 1.8 fold in glucose containing medium. Alpha glucosidase is an enzyme which hydrolyzes sucrose for sucrose utilization and is induced by maltose and suppressed by glucose. In 2006, Bramono *et al*.^66^ reported that cell surface alpha-glucosidase activity was very high in *C. tropicalis, C. albicans* and *C. parapsilosis* than other *Candida* species. Recently, Kim and his colleagues (2018)^67^ reported that Magnoflorine compound could inhibit the alpha-glucosidase activity (>50%) in *C. albicans* which is essential for normal cell wall composition and virulence.

Thiamine dependent enzyme, transketolase (Tkl1p), an important enzyme of carbon metabolism, is up regulated in response to cadmium, salt stress and hydrogen peroxide^68^. In recent years targeting thiamine biosynthetic pathways has emerged as the new strategy for the development of antifungal drugs (Meir and Osherov, 2018)^69^. Also, Siemieniuk, *et al*.^70^ reported the inhibitory effect of thiamine antivitamins on the growth and metabolism of pathogenic *C. albicans* and *M. pachydermatis*. In the present study, down regulation in the expression of Thiamine dependent enzyme, transketolase (Tkl1p), suggests that oleic acid may possibly target the thiamine biosynthetic pathway for its antivirulence property.

NADPH-dependent diflavin oxidoreductase 1 (Tah18p) is the part of iron-sulfur (Fe-S) cluster assembly, required for the maturation of extra mitochondrial Fe-S protein. In *C. albicans*, iron is an important micronutrient which controls its pathogenicity where survival of this pathogen in host depends on iron homeostasis by regulating iron uptake and storage^71^. In addition, *C. albicans* consist of tripartite system of transcription factors such as Sef1 (Zn_2_Cys_6_ DNA-binding protein), Sfu1 (GATA factor), and Hap43 (CCAAT binding complex) to control iron homeostasis^72^. These transcriptional regulators (Sef1 and Rim101) activate the virulence genes expression during iron deprivation. In the present study, oleic acid down regulated the Tah18p, thereby inactivating the Fe-S assembly and downregulated the expression of virulence factors.

Mitochondrial import inner membrane translocase subunit Tim16 (Pam16) is a part of PAM complex which is involved in the ATP-dependent translocation of the transit peptide containing proteins. In addition to that, *PAM16* gene is involved in carbohydrate metabolism and up regulated during intermediate and mature biofilm development stages^73^. In the current study, oleic acid inhibited mature biofilm formation without affecting metabolic viability, which is also reflected on the down regulation of Pam16p (1.6 fold).

Squalene synthase (Erg9) enzyme catalyzes the reduction of two farnesyl pyrophosphate into squalene in ergosterol biosynthesis pathway. Ergosterol is the well-known target for many antifungal agents and depletion in ergosterol content affects major cellular processes and membrane properties^74^. Moreover, farnesol (200 μM) is the quorum sensing signaling molecule produced by *Candida* spp., which down regulates the expression of *ERG9*, *ERG20* and *ERG11*^75^. Similarly, bafilomycin C1 compound, isolated from *Streptomyces albolongus*, down regulates the expression of Erg9 and other genes involved in ergosterol biosynthetic pathway^76^. In the present study, oleic acid down regulated the expression of Erg9p by 1.5 fold and this result was further substantiated by *in vitro* quantification of ergosterol and qPCR analysis.

In *C. albicans*, secreted lipase gene family contains 10 members (*LIP1-LIP10*)^77^. Hydrolytic enzymes such as SAPs, phospholipases and lipases are considered as important virulence factors of *C. albicans* which are responsible for the cell membrane damage in host and thereby promoting adhesion, invasion and colonization^78^. Down regulation of Lip1p (1.5 fold) by oleic acid treatment was further validated by *in vitro* experiment for lipase production. Likewise, the farnesol related compound, cis-2-dodecenoic acid decreased the expression of *LIP1* (36.2%) of *C. albicans*^79^.

Peroxiredoxin (Tsa1B) is a thiol-specific peroxidase that catalyzes the reduction of hydrogen peroxide to water and organic hydroperoxides to alcohol. Tsa1p is differentially localized in *C. albicans*, wherein cell wall of hyphae and nucleus of yeast and Tsa1p are essential for the proper organization of cell wall and provide resistance to oxidative stress^80,81^. In *C. albicans*, two cytosolic Mn Superoxide Dismutases (SOD1 and SOD3) and one mitochondrial CuZn SOD (SOD2) prevent the cells from ROS^43^. H_2_O_2_ sensitivity assay results confirmed that increasing concentration of oleic acid (40, 80 and 160 μg mL^−1^) treated *Candida* cells are more sensitive to H_2_O_2._ Inhibitory effect of oleic acid on yeast-to-hyphal transition and oxidative stress tolerance might be the reason for the down regulation of Tsa1b (1.5 fold). This result goes in parallel with our previous report wherein higher concentration of myristic acid (125 and 250 μg mL^−1^) sensitizes the *C. albicans* to H_2_O_2_^43^_._

In the present study, up regulation (3.0 fold) of Rex2p was observed in *C. albicans* upon oleic acid treatment. Rex2p (orf19.1466) was found to be involved in transcription (3’-5’-exoribonuclease activity) nucleic acid binding and ribosome biogenesis in eukaryotes^82^. Up regulation of Rex2p suggests that oleic acid did not affect the transcription and ribosome binding processes. The next important protein up regulated (1.9 fold) in the present study was serine hydroxymethyltransferase (Shm2) which catalyzes the interconversion of serine and glycine. Glycine is a small amino acid which plays a crucial role in one carbon metabolism thus promoting cell division^83,84^. In the present study, oleic acid did not affect amino acid biosynthesis of *C. albicans* and that might be the reason for the up regulation of Shm2p. Another up regulated protein Hexokinase-2 (1.9 fold) is a well-known phosphorylating enzyme that initiates glycolysis metabolic pathway by converting glucose to glucose 6-phosphate. Upregulation of Rex2p, Shmp and Hxk2p further substantiates the non-antimicrobial effect of oleic acid against *C. albicans* which has also been corroborated through antimicrobial assay and XTT assay.

**Figure 8:**
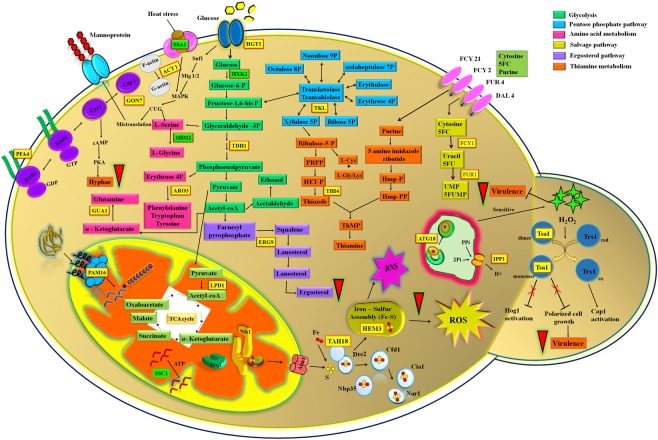
Schematic representation of the proteins and pathways of *C. albicans* embattled by oleic acid. Up regulated and down regulated protein spots are highlighted in green and yellow boxes, respectively.

Heat shock proteins (Hsps) exist in most of the organisms and protect the organism from biotic and abiotic stresses^85^. In *C. albicans*, Hsps are found to be involved in MAPK, Ras1-cAMP-PKA, calcium-calcineurin, and cell cycle control signaling pathways. Recent studies reported that Hsps (Hsp70 and Hsp90) inhibit normal growth of *C. albicans* and thereby confer drug resistance to the pathogen^86^. Moreover, Sapiro *et al*.^87^ reported that pharmacological inhibition of Hsp90 by geldanamycin induced the filamentous growth of *C. albicans* in liquid rich medium at 30 °C. Consequently, in the present study, oleic acid exhibits significant inhibition on hyphal growth of *C. albicans* and that might be the reason for the up regulation of heat shock protein Ssa1 or Hsp70 (1.6 fold). Yet another Hsp up regulated in the present study was mitochondrial heat shock protein Ssc1, which is involved in protein transport in mitochondria. Besides, Ssc1p expression was up regulated in electron transport chain complex I mutants (*nuo1Δ* and *nuo2Δ*) which responds to amino acid starvation^88^. Hence, Hsps and Hsps associated pathways are seen as novel antifungal targets against candidiasis treatment. In the present study, up regulation of Ssc1p (1.5 fold) was observed which suggests that oleic acid did not affect the mitochondrial protein import, protein folding and energy metabolism. Schematic representation of differentially regulated proteins and pathways targeted by oleic acid in *C. albicans* is depicted in Fig. 8.

On the whole, the present study reports the inhibitory efficacy of oleic acid on *C. albicans* virulence including biofilm formation. *In vitro* virulence assays revealed the inhibitory effect of oleic acid on *Candida* spp. biofilm development, morphological transition, secreted hydrolases production and ergosterol biosynthesis. These results were further substantiated by gene expression studies. Further, proteomic analysis of *C. albicans* revealed that oleic acid exerts stress conditions such as heat stress, ROS and RNS and also targets proteins involved in basic pathways such as glucose metabolism, nucleic acid, amino acid and vitamin biosynthesis. Hence, these proteins are considered as highly conserved and they are speculated to be the key players involved in anti-virulence activity of oleic acid in *C. albicans*. Thus, the present study suggests that oleic acid could be used as an ideal agent for the treatment of biofilm associated infection caused by *Candida* spp.

## Methods

### Ethics statement

The present study was carried out in reference to the recommendations of Ethical Guidelines for Biomedical Research on Human Subjects, issued by Indian Council of Medical Research. The protocol was approved by the Institutional Ethics Committee, Alagappa University (Ref No: IEC/AU/2014/2). All participants gave written informed approval in accordance with the Declaration of Helsinki.

### Fungal strains and growth conditions

In this study, *C. albicans* ATCC 90028, *C. glabrata* MTCC3019, *C. tropicalis* MTCC 184, *C. albicans* clinical isolates CA1 (MF423465), CA2, CA3 (MF423466), CA4 (MF423467) and *C. tropicalis* clinical isolates CT1 (MF423462), CT2 (MF423463), CT3 (MF423464) were used. The clinical isolates were collected from patients with *Candida* infection, identified at the species level by ITS sequencing. All the strains were maintained on Sabouraud dextrose agar (SDA) (Himedia, India) plates at 4 °C. To determine the effect of oleic acid on *Candida* spp. growth and biofilm, YEPD (1% Yeast extract, 2% glucose, and 2% Peptone) and spider broth (1% Mannitol, 0.2% K_2_HPO_4,_ and 1% Nutrient broth) were used, respectively. For filamentation assay, spider agar was used. All the culture media components used in the present study were procured from Himedia, India and all the reagents used for the proteomics study were procured from Sigma, USA.

### Antibiofilm assay and microscopic observation of biofilms

In order to check the antibiofilm efficacy of oleic acid on *Candida* spp., biofilm susceptibility assay was performed using the method as previously described by our group^17^. In brief, *Candida* spp. biofilm was grown in spider broth in the absence and presence of oleic acid (at different concentrations 5, 10, 20, 40, 80, 160 and 320 μg mL^−1^) in 24 well MTP at 37 °C for 24 h. After incubation, loosely adherent planktonic cells were washed with distilled water and the sessile biofilm cells were stained with 0.4% crystal violet for 5 min followed by washing off the excess stain with distilled water. Finally, 1 mL of glacial acetic acid was used to dissolve the crystal violet present in biofilm cells and absorbance was measured at 570 nm. Biofilms formed on glass surfaces in the presence and absence of oleic acid were visualized under light and confocal laser scanning microscopes. Detailed procedures are provided in Supplementary Data.

### Antimicrobial assay and XTT assay

To determine the antifungal activity of oleic acid at BIC against *Candida* spp. growth, absorbance at 600 nm was measured using multifunctional spectrometer. In addition, XTT reduction assay was performed to assess the effect of oleic acid on *Candida* spp. cell viability^15^. Detailed procedures are provided in Supplementary Data.

### Filamentation assay

The effect of oleic acid on *Candida* spp. filamentation was assessed using spider agar medium containing 1% fetal bovine serum (FBS) and the filamentous growth of *Candida* spp. were photographed. Detailed procedures are provided in Supplementary Data.

### EPS extraction and quantification

EPS was extracted from oleic acid treated and untreated *Candida* strains by the method followed by our previous study^15^ with slight changes. Total carbohydrates, lipids, protein and eDNA level in EPS were quantified using phenol sulfuric acid method (optical density (OD) at 490 nm), phospho-vanillin method (OD at 545 nm), Bradford method (OD at 595 nm) and nano spectrophotometer (OD at 260/280 ratio), respectively. Detailed procedures are provided in Supplementary Data.

### Quantification of SAPs and lipases

SAPs and lipase production was qualitatively measured using bovine serum albumin (BSA) agar and tributyrin agar, respectively. White opaque zone around the colonies represent SAPs production and zone of clearance around the colonies indicate lipase production. Detailed procedures are provided in Supplementary Data.

### Ergosterol extraction

Changes in the ergosterol content in the absence and presence of oleic acid was measured spectrometrically^3^. Ergosterol was extracted from the control and oleic acid treated *Candida* spp. and scanned spectrophotometrically between 200 and 300 nm. Detailed procedures are provided in Supplementary Data.

### Adhesion assay

Alamar blue assay was performed to assess the adhesion ability of *C. albicans* on polystyrene surfaces in the absence and presence of oleic acid at different concentration (10, 20, 40, 80 and 160 μg mL^−1^). The control and oleic acid treated cells were incubated with alamar blue solution and the fluorescent intensity was measured at 590 nm emission and 560 nm excitation wavelengths^89^. Detailed procedures are provided in Supplementary Data.

### H_2_O_2_ sensitivity assay

Overnight grown *C. albicans* culture in the presence and absence of oleic acid (80 and 160 μg mL^−1^) OD_600 nm_ was adjusted to 0.3 and swabbed on YEPD agar plates. To assess the H_2_O_2_ sensitivity, sterile filter paper disks with 10 mm diameter (Himedia, India) were placed on YEPD agar plates, loaded with 15 μL of 30% H2O2 and incubated at 37 °C for 16 h. After incubation, zone of clearance was measured and the plates were documented^43^.

### Real Time PCR

Total RNA was isolated from control and oleic acid treated (at BIC) cultures using hot phenol extraction method^15^ and converted into cDNA using High capacity cDNA Reverse Transcription kit (Applied Biosystems, USA). Candidate virulence genes (*als1*, *als3*, *cdr1*, *mdr1*, *erg11*, *flu1*,*nrg1, sap1, sap2, sap4*, *tup1*, *hwp1*, *eap1*, *efg1*, *cst20*, *ras1*, *ume6*, *hst7* and *cph1, chs3, cht4*,) were selected for real time PCR experiment (7500 Sequence Detection System, Applied Biosystems Inc. Foster, CA, USA). The primers were combined individually with SYBR Green kit (Applied Biosystems, USA) at a predefined ratio. The expression pattern of candidate genes were normalized against ITS gene (~540 bp) expression (housekeeping gene) and quantified using the ΔΔCT method. Detailed procedures are provided in Supplementary Data.

### Intracellular protein extraction

For intracellular protein extraction, *C. albicans* was grown in the absence and presence of oleic acid (80 μg mL^−1^) in YEPD supplemented with FBS and incubated at 37 °C in a shaking condition for 16 h (until the cells reach mid log phase). Then, the cells were collected, by centrifugation (8000 rpm, 20 min at 4 °C) and washed twice with PBS (pH – 7.4) and sonicated in 20 mM Tris-HCl (pH – 8.0) containing 1% protease inhibitor cocktail, 100 mM PMSF and 1 mM of 0.5 M EDTA. After sonication, cell suspension was centrifuged (13000 rpm, 30 min at 4 °C) and the supernatant was collected. To purify the cellular proteins, equal volume of phenol was added to the supernatant, and incubated at 70 °C for 10 min and cooled at 4 °C for 10 min. Then, equal volume of milli Q water was added to the phenol/protein extract mixture, vortexed and incubated as mentioned in the previous step. Subsequently, the tubes were centrifuged (8,000 rpm, 10 min at 4 °C) for phase separation. The aqueous phase was discarded; protein present in the phenol phase was precipitated with double the volume of ice cold acetone. Then, the precipitated proteins were collected by centrifugation (12,000 rpm, 15 min at 4 °C) and washed thrice with acetone to remove the residual phenol. After thrice washing with acetone, the pellets air dried. Then, the dried protein pellets were dissolved in sample buffer containing 7 M Urea, 2 M thiourea and 4% CHAPS^11^.

### Two dimensional gel electrophoresis

Two dimensional gel electrophoresis (2DGE) was performed using the method described in our previous report^11^. Briefly, first dimension/isoelectric focusing (IEF) was performed in IPGphor 3 system using immobiline DryStrip gel strips (18 cm, non- linear, pH 4–7). Protein samples (each 1000 μg from control and oleic acid treated) were mixed with rehydration buffer containing 7 M urea, 2 M thiourea, 2% (CHAPS), 12.5 mg mL^−1^ destreak reagent and 0.5% IPG buffer (pH 4–7) to a final volume of 350 μL and applied to the IPG strips by in- gel rehydration for 16 h at 20 °C. After rehydration, IPG strips containing protein samples were subjected to isoelectric focusing at 20 °C under mineral oil using following conditions: 2 h at 100 V; 3 h at 500 V (gradient); 3 h at 500 V; 2.5 h at 5000 V (gradient); 2 h at 5000 V (gradient); 3 h at 8000 V (gradient); 2 h at 8000 V and final focussing was done for 2 h at 10000 V. The current was set to 75 μA per IPG strip. After IEF, the IPG strips were incubated in equilibration buffer I [6 M urea, 30% (w/v) glycerol, 2% (w/v) sodium dodecyl sulphate (SDS) and 1% (w/v) DTT in 50 mM Tris-HCl buffer, pH 8.8] followed by equilibration buffer II [6 M urea, 30% (w/v) glycerol, 2% (w/v) SDS and 2.5% (w/v) iodacetamide (IAA) in 50 mM Tris-HCl buffer, pH 8.8] for 15 min each. After two equilibration steps, for second dimension, IPG strips were placed on 22 cm × 22 cm × 1 mm 10–15% gradient sodium dodecyl sulphate- polyacrylamide gels (SDS-PAGE) and overlaid with 0.3% agarose. Electrophoresis was performed at 100 V for 1 h and 150 V for 8 h in Ettan DALT six apparatus (GE Healthcare, USA). After electrophoresis, gels were immersed in fixative for 3 h and washed thrice with milli Q water for 20 min each. Protein spots were stained using CBB G-250 staining solution for 12 h on rotary agitator. After staining, gels were destained with milli Q water for 4 h to reduce the background^11^.

### Protein spot excision and trypsin digestion

Differentially regulated protein spots with more than 1.5 fold changes in the intensity (both up and down regulated) were selected and excised from the gels for protein identification. Briefly, prior to destaining, gel pieces were washed with milli Q water. Then, the gel pieces were completely destained by washing with destaining solution containing 50% acetonitrile containing 25 mM ammonium bicarbonate (NH_4_HCO_3_). Destained gel pieces were completely dehydrated in 100% acetonitrile (ACN) for 10 min and dried under vacuum for 30 min. Dried gel pieces were incubated in reduction solution (10 mM DTT and 25 mM NH_4_HCO_3_) followed by alkylation solution (25 mM NH_4_HCO_3_ and 55 mM IAA) for 30 min each. After reduction and alkylation step, the gel pieces were dehydrated with 100 μL of acetonitrile and dried under vacuum for 30 min. Then, the gel pieces were rehydrated/trypsinized with 5 μL of digestion buffer (10 mM NH_4_HCO_3_ in 10% ACN) containing 400 ng of trypsin (Sigma Aldrich) on ice for 30 min. Then, the rehydrated gel pieces were covered with 25 μL of overlay buffer (40 mM NH_4_HCO_3_ in 10% ACN) and incubated at 37 °C for 16 h. After incubation, peptides were extracted twice with 25 μl of 0.1% trifluoroacetic acid (TFA) in 60% ACN by sonication (10 min) followed by 20 μL of 100% ACN. Extracted peptides were dried under vacuum for 90 min and stored at 4 °C^11^. Before MALDI-TOF/TOF analysis, peptides were dissolved in peptide resuspension solution containing 0.1% TFA in 5% ACN. Then, the salts present in the peptides were removed using C18 zip tips (Merck Millipore), the desalted peptides were dried and stored at 4 °C.

### MALDI- TOF/TOF analysis

Differentially regulated proteins upon oleic acid treatment were analyzed using MALDI- TOF/TOF analysis^13^. Briefly, equal volume (1 μL) of peptides were mixed with 1 μL of matrix solution [alpha-cyano-4-hydroxy cinnamic acid matrix (10 mg mL^−1^), acetonitrile (50%) and trifluroacetic acid (0.1%)] and spotted on MALDI target plate. The MALDI- TOF/TOF mass spectrometer (AXIMA Performance, SHIMADZU BIOTECH) with laser wavelength of 337 nm was calibrated using TOF- Mix™ (LaserBio Labs, France). Peptide mass spectra were acquired from a MALDI- TOF/TOF mass spectrometer in positive reflector mode and analysed by Shimadzu launch pad- MALDI MS software (Version- 2.9.3.20110624, Kratos Analytical Ltd, U.K.). For single spot, 45 subspectra resulted after 50 laser shots at randomized positions with total of 2250 laser shots. The laser intensity for MS spectrum acquisition was 4500. Mono isotopic peak list (m/z range of 700–4000 Da with S/N ratio over 10) generated by MALDI MS software was analyzed using online software tool MS-Fit from Protein Prospector (v 5.24.0). (http://prospector.ucsf.edu/prospector/cgi-bin/msform.cgi?form=msfitstandard) with the standard protein identification parameters. Briefly, a mass tolerance of 1.50 Da per peptide, maximum of 2 missed cleavages per peptide, carbamidomethylation of cysteine was given as fixed modification whereas N-terminal acetylation and phosphorylation (S, T, Y) and methionine oxidation, as variable modifications.

### Statistical analysis

All the experiments were performed at least twice in independent experiments in triplicates to confirm reproducibility and the data were presented as mean ± standard deviation. For all experiments, statistical differences between control and treated samples were analyzed with one way ANOVA followed by Dunnett’s test with significant p-value <0.05 using SPSS software version 17.0 (Chicago, IL, USA).

## Supplementary information

Supplementary information

## Data Availability

The raw data of MALDI-TOF/TOF was submitted in ProteomeXchange Consortium via the PRIDE partner repository with the dataset identifier PXD017588.
