# Supplementary material for: Global proteomic analysis deciphers the mechanism of action of plant derived oleic acid against Candida albicans virulence and biofilm formation
Source: Sci Rep. 2020 Mar 20;10:5113. doi: 10.1038/s41598-020-61918-y (PMC7083969; doi:10.1038/s41598-020-61918-y)

**Global proteomic analysis deciphers the mechanism of action of plant derived oleic acid against *Candida albicans* virulence and biofilm formation**

Subramanian Muthamil^†1^, Krishnan Ganesh Prasath^†1^, Arumugam Priya^1^, Pitchai Precilla^1^ and Shunmugiah Karutha Pandian*^1^

†Equal contribution

^1^ Department of Biotechnology

Science Campus

Alagappa University

Karaikudi – 630 003

Tamil Nadu

India

* Corresponding author.

Shunmugiah Karutha Pandian

Senior Professor and Head

Department of Biotechnology

Science Campus

Alagappa University

Karaikudi – 630 003

Tamil Nadu

India

E. mail: sk_pandian@rediffmail.com

Tel: + 91 4565 225215

Fax: + 91 4565 225202

**Supplementary Methods**

**Microscopic analysis of biofilm**

For microscopic observation, *Candida* spp. biofilm was grown on 1 x 1 cm glass slide in spider broth for 24 h with and without oleic acid (at BIC). Then, the slides were washed with sterile distilled water and air dried. For light microscopy, o.4 % crystal violet was used to stain the biofilm cells, washed with distilled water and kept for air dry. Then, the stained glass pieces were visualized under light microscope (Nikon Eclipse 80i, Japan) at 400x magnification. For CLSM analysis, instead of crystal violet, 0.1 % acrydine orange was used for staining and kept at dark for 5 min. Then the slides were washed with distilled water, air dried and observed under CLSM (Carl Zeiss LSM710, Germany) at 200x magnification.

**Antimicrobial assay**

To determine the antifungal activity of oleic acid against *Candida* spp. growth, micro broth dilution assay was performed in 24 well MTP^21^. In 1 mL of YEPD broth, oleic acid was added at different concentrations (10 µg mL^-1^ to 640 µg mL^-1^). All the 10 Candida strains (~10^7^ cells) were separately used as inoculum for each well and incubated at 37 °C for 24 h. Sterile YEPD broth inoculated with Candida cell and sterile broth alone served as control and blank, respectively. After incubation, absorbance at 600 nm was measured using multifunctional spectrometer.

**XTT assay**

XTT reduction assay was performed to assess the effect of oleic acid on *Candida* spp. cell viability.^15^ In brief, equal amount of cells were used to inoculate 1 mL of spider broth in the absence and presence of oleic acid (at BIC) and incubated at 37 °C for 24 h. After incubation, both oleic acid treated and untreated cells were washed twice with sterile PBS and resuspended in the 100 μL of the same. Just prior to the experiment, XTT-Menadione solution was freshly prepared at the ratio of 12.5:1. Twenty five microlitre of XTT-Menadione mixture was added to oleic acid untreated and treated cell suspension and incubated at 37 °C for 4 h. Then, cells were removed by centrifugation and the absorbance of supernatant was calculated. Sterile PBS plus XTT-Menadione solution served as blank.

**Filamentation assay**

The effect of oleic acid on *Candida* spp. filamentation was assessed using spider agar medium containing 1 % fetal bovine serum (FBS) was used^15^. Five microlitre of 24 h old *Candida* spp. with and without oleic acid (at BIC) was spotted on the agar surface and incubated at 37 °C for 3 days. After incubation, the images with filamentous growth of *Candida* spp. were captured using gel documentation system (GelDoc XR+, Bio-Rad, USA).

**EPS extraction and quantification**

EPS was extracted from QA-UDA combination treated and untreated Candida strains by the method followed by our previous study^15^ with slight changes. Briefly, 10 mL of *Candida* spp. culture grown in the absence and presence of oleic acid (at BIC) was centrifuged and the cells were collected and the supernatents were stoed at -80 °C. Cell pellet was washed with sterile PBS and suspended in 10 mL of isotonic buffer (10 mM Tris/HCl pH 8.0, 10 mM EDTA, 2.5% NaCl) and incubated at 4 °C for 12 h. After incubation, isotonic buffer containing the cell suspension was vortexed for 3 min and centrifuged at 10000 rpm for 15 min and the supernatant was mixed with CFCS. Then, both cell bound and secreted EPS were precipitated by ice cold ethanol and incubated at -20 °C for 12 h. Finally, pelleted form of EPS was collected by centrifugation at 10000 rpm for 15 min, and dried in rotary vacuum evaporator (Christ Alpha 2-4 LD plus, UK). For the EPS quantification, both control and oleic acid treated EPS samples were dissolved using distilled water. Total carbohydrates, lipids, protein and eDNA level in EPS were quantified using phenol sulfuric acid method (optical density (OD) at 490 nm), phospho-vanillin method (OD at 545 nm), Bradford method (OD at 595 nm) and nano spectrophotometer (OD at 260/280 ratio), respectively. Inhibition of EPS components was calculated using the formula:

% of inhibition = [(Control OD– Treated OD)/ Control OD] x 100

**Quantification of SAPs and lipases**

**(i) SAPs**

SAPs production was qualitatively measured by the method described in our previous study^15^ using bovine serum albumin (BSA) agar (1 % Glucose, 0.05% MgSO4, 2% agar, 1% BSA, pH - 4.5). The 24 h old *Candida* spp. culture (5 µL) with and without oleic acid (at BIC) was spotted on the top of the agar and incubated at 37 °C for 3 days. After incubation, white opaque zone around the colonies representing the SAPs production and zone diameter was measured using Hiantibiotic zone scale (Himedia, Mumbai).

**(ii) Lipases**

The effect of oleic acid on *Candida* spp. lipase production was determined using tributyrin agar (0.8 % peptone, 0.4 % yeast extract, 0.3 % NaCl, 2.0 % Agar and after autoclaving 0.2 % tributyrin)^15^. Five microlitre of oleic acid treated and untreated *Candida* spp. culture (24 h old) was spotted at the centre of agar surface and incubated at 37 °C for 48 h. After incubation, zone of clearance around colonies indicating lipase production was measured by Hiantibiotic zone scale.

**Ergosterol extraction**

Changes in the ergosterol content in the absence and presence of oleic acid was measured by UV spectrophotometer^3^. A 24 h grown *Candida* spp. culture was used to inoculate 10 mL of YEPD supplemented with oleic acid (at BIC) and incubated at 37 °C for 24 h. Then, the cells were collected by centrifugation, washed once with distilled water, weight measured and the cells were suspended in 25 % alcoholic potassium hydroxide and vortexed for 1 min. Afterwards, the tubes were incubated at 80 °C for 1 h and cooled at room temperature. Then, the sterol was extracted with 1mL of distilled water and 3 mL of n-heptane. The mixture was vortexed continuously for 10 min i.e., until the distinct layer of n-heptane was noticed. The heptane layer was transferred to a clean borosilical tube. Further, sterol extract of 20 μL was diluted up to 5-fold using absolute ethanol and scanned spectrophotometrically between 200 and 300 nm with a UV spectrophotometer (UV 2450, Shimadzu, Japan).

**Adhesion assay**

Alamar blue assay was performed to assess the adhesion ability of *C. albicans* on polystyrene surfaces in the absence and presence of oleic acid at different concentration (10, 20, 40, 80 and 160 μg mL^-1^). Prior to experiment, stock solution of Alamar blue (Sigma-Aldrich, India) at the concentration of 6.5 mg mL^-1^ in Phosphate buffered saline (PBS) (1X) was prepared separately. *C albicans* biofilm was grown in spider broth in the absence and presence of oleic acid (at different concentrations 10, 20, 40, 80, 160 μg mL^-1^) in 24 well MTP at 37 °C for 24 h. After incubation, slackly attached planktonic cells were discarded and the sessile biofilm cells were washed twice with PBS and resuspended in the same. To the 0.9 mL of cell suspension, 0.1 mL of alamar blue substrate was added and incubated in the dark at 37°C for 4 h. Sterile PBS along with Alamar blue substrate was kept as blank. After incubation, the samples were centrifuged at 8000 rpm for 10 min. The fluorescent intensity of the supernatant containing the reduced Alamar blue was measured at 590 nm emission and 560 nm excitation wavelengths.

**Real Time PCR**

The effect of oleic acid on the gene expression of important virulence factors of *C. albicans* was assessed using real time PCR (Applied Biosystems, USA). For this, total RNA was isolated from control and oleic acid treated (at BIC) cultures using hot phenol extraction method^15^. Then, cDNA was synthesized from isolated RNA samples using High capacity cDNA Reverse Transcription kit (Applied Biosystems, USA). Candidate virulence genes (*als1*, *als3*, *cdr1*, *mdr1*, *erg11*, *flu1*,*nrg1, sap1,* *sap2, sap4*, *tup1*, *hwp1*, *eap1*, *efg1*, *cst20*, *ras1*, *ume6*, *hst7*, *cph1, chs3* and *cht4*) were selected for real time PCR experiment (7500 Sequence Detection System. The primers were combined individually with SYBR Green kit (Applied Biosystems, USA) at a predefined ratio. The PCR cycle had the temperature pattern of initial denaturation at 94 °C for 10 min, denaturation at 94 °C for 1 min, annealing at 55 °C & 60 °C for 1 min and extension at 72 °C. The expression pattern of candidate genes were normalized against ITS gene (~540bp) expression (housekeeping gene) and quantified using the ^ΔΔ^CT method.

**Supplementary Figure**

**Supplementary figure S1** **(a)** Effect of oleic acid on growth of *Candida* spp. in YEPD broth at 37 ºC for 24 h. No antifungal activity was observed at tested concentrations (10 µg mL^-1^ to 640 µg mL^-1^) in all the tested *Candida* strains. **(b)** Effect of oleic acid on *Candida*
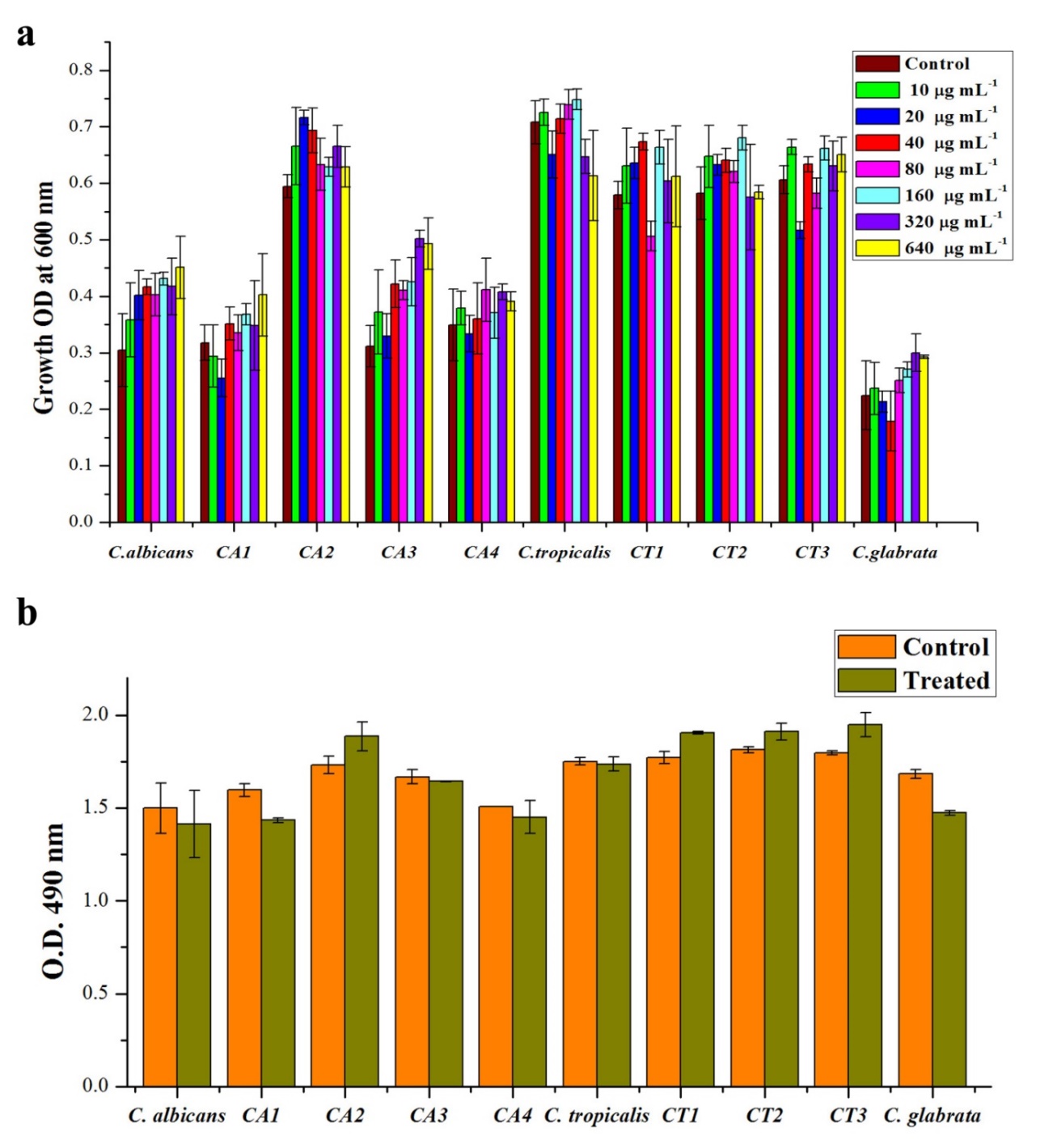
 spp. metabolic viability. No significant change was observed in the cell viability of tested Candida strains when compared to untreated controls. Error bars represent standard deviations from the mean (n=3). Statistical significance was analyzed using one way ANOVA-Duncan’s post-hoc test and asterisk represents p<0.05.

**Uncropped Gel Images**

**Figure 5: Effect of oleic acid on intracellular proteome of *C. albicans***

Representative gel image of intracellular protein of *C. albicans* without treatment


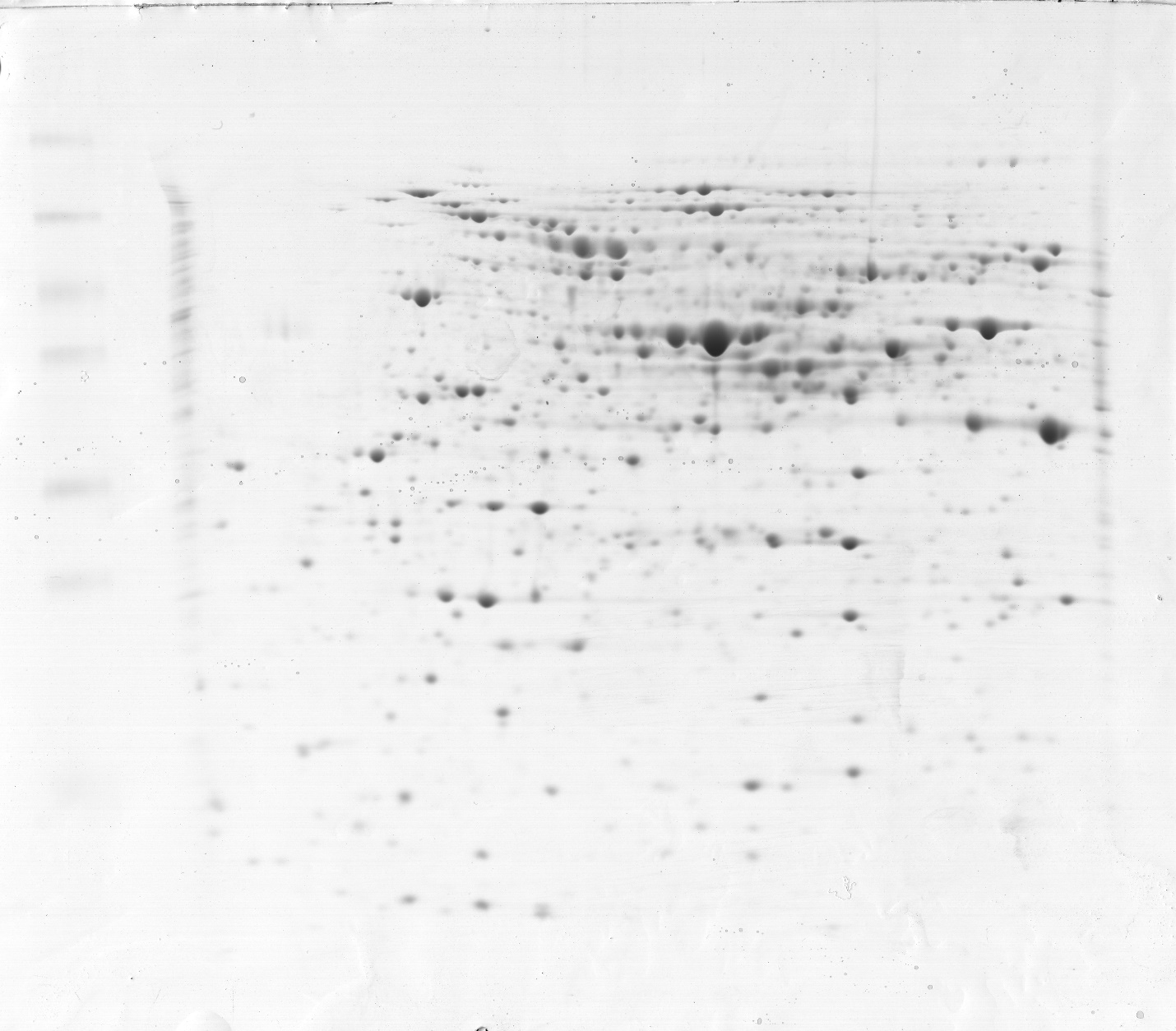


Representative gel image of intracellular protein of *C. albicans* treated with oleic acid.


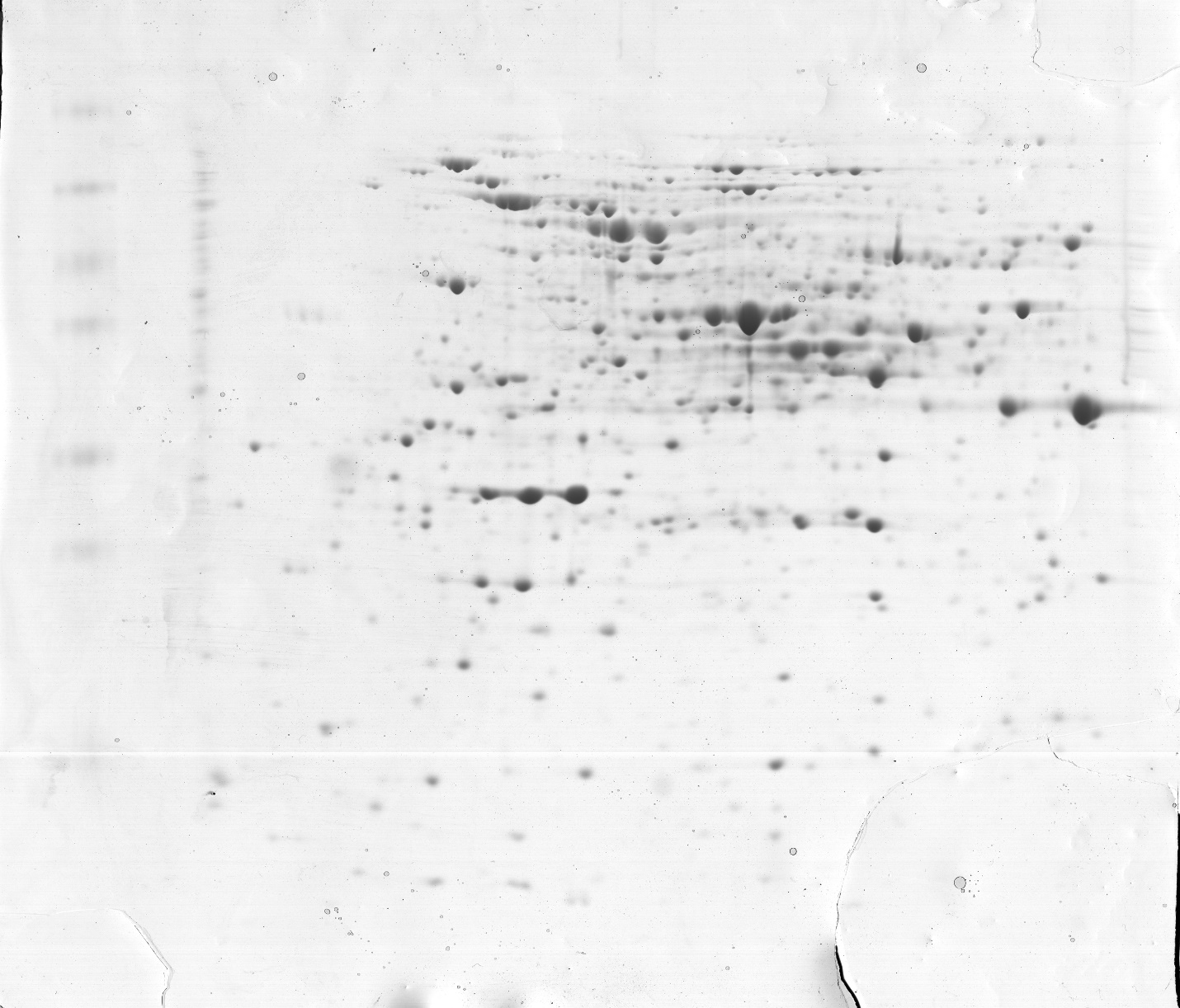

Supplement: Supplementary file 1 — Supplementary information [file 41598_2020_61918_MOESM1_ESM.doc]
